# Genotype-Specific Photosynthetic Plasticity and Leaf Yield of *Stevia rebaudiana* Under Contrasting Radiation Across Caribbean Environments

**DOI:** 10.3390/plants15060896

**Published:** 2026-03-13

**Authors:** Alfredo Jarma-Orozco, Anthony Ariza-González, Juan Jaraba-Navas, Enrique Combatt-Caballero, Luis Alfonso Rodríguez-Páez

**Affiliations:** Facultad de Ciencias Agrícolas, Universidad de Córdoba, Montería 230002, Colombia; ajarma@correo.unicordoba.edu.co (A.J.-O.); aarizagonzalez28@correo.unicordoba.edu.co (A.A.-G.); jjaraba@correo.unicordoba.edu.co (J.J.-N.); emcombatt@correo.unicordoba.edu.co (E.C.-C.)

**Keywords:** chlorophyll fluorescence, gas exchange, genotype × environment interaction, photosynthesis, photosynthetic photon flux density, stomatal conductance

## Abstract

Light availability drives *Stevia rebaudiana* productivity, yet how incident radiation interacts with genotype and site under tropical field conditions remains unclear. We evaluated four genotypes (L020, L102, L082, and ‘Morita II’) across three Caribbean locations in Colombia under two contrasting light levels (600 vs. 1800 μmol photons m^−2^ s^−1^) using a split-plot randomised complete block design with four replicates. Incident photosynthetic photon flux density (PPFD) was logged and, at 85 days after transplanting (DAT), net CO_2_ assimilation, stomatal conductance, transpiration, and intercellular CO_2_ concentration were measured alongside light-adapted chlorophyll fluorescence parameters, including the effective quantum yield of photosystem II (ΦPSII), the maximum efficiency of PSII in the light (Fv′/Fm′), photochemical quenching (*qP*), and electron transport rate (ETR); biomass and leaf yield were quantified at harvest. Data were analysed using factorial analysis of variance (ANOVA) and complementary multivariate approaches, including Pearson correlation analysis and principal component analysis (PCA). Radiation responses were strongly site-dependent: under 1800 μmol photons m^−2^ s^−1^, net CO_2_ assimilation increased by 90.2% at El Carmen de Bolívar and 21.5% at Polonuevo but decreased by 36.4% at Montería. Leaf yield was highest in El Carmen de Bolívar (1951.46 ± 182.03 kg ha^−1^), followed by Montería (1510.94 ± 173.75 kg ha^−1^) and Polonuevo (576.31 ± 42.36 kg ha^−1^). Genotype rankings shifted with environment and radiation, with L102 reaching 3256.25 ± 126.39 kg ha^−1^ under direct radiation in El Carmen de Bolívar and ‘Morita II’ showing strong responsiveness in Montería. These results demonstrate that photosynthetic plasticity and leaf yield in *S. rebaudiana* depend on genotype × radiation × environment interactions, supporting location-tailored radiation management combined with targeted genotype deployment.

## 1. Introduction

Reducing free-sugar intake and reformulating processed foods are now central pillars of global public-health agendas, accelerating the deployment of high-intensity, non-sugar sweeteners across beverage and food systems. Among the few plant-derived options with broad regulatory acceptance, steviol glycosides from *Stevia rebaudiana* stand out because they deliver high sweetness potency with negligible caloric contribution, aligning with the current transition toward healthier dietary patterns and “clean-label” product portfolios [1,2,3].

However, as *S. rebaudiana* cultivation expands beyond its traditional subtropical niches into lowland tropical agroecosystems, productivity is increasingly constrained by a coupled radiative–atmospheric stress regime: very high midday irradiance coinciding with high vapour pressure deficit (VPD). Elevated VPD can rapidly depress stomatal conductance (*g_s_*), restricting CO_2_ supply to the chloroplast and reducing net photosynthesis (A), while excess photon flux simultaneously increases excitation pressure on the photosystems—conditions that favour down-regulation of photochemistry and greater reliance on photoprotective energy dissipation [4,5]. In practice, this means that, under tropical field conditions, “good light” can become non-usable light unless plants can maintain coordinated stomatal and photochemical regulation across the diel cycle.

Mechanistically, the limitation is not only “how much” light is available, but how fast the photosynthetic apparatus can track rapid changes in irradiance under a tight water–energy balance. Slow stomatal kinetics under fluctuating light can impose substantial penalties on carbon gain and intrinsic water-use efficiency, particularly when high VPD pushes stomata toward conservative behaviour [6]. At the same time, chlorophyll fluorescence metrics provide sensitive, field-deployable diagnostics of PSII operating efficiency (e.g., ΦPSII), electron transport (ETR proxies), and regulated energy dissipation (non-photochemical quenching (NPQ)), enabling a quantitative readout of how leaves partition absorbed light between photochemistry and photoprotection in situ [7]. Together, leaf gas exchange (*A_N_*, *g_s_*) and chlorophyll fluorescence capture the two core control points of tropical performance: CO_2_ diffusion and photochemical energy use.

From an agronomic standpoint, incident-radiation management is one of the few scalable levers available to buffer this stress couplet in open-field and semi-protected systems. Shade nets and related light-modifying materials can reduce peak irradiance, lower leaf temperature, and alter canopy light distribution, frequently improving gas exchange and productivity in horticultural crops when heat and evaporative demand are limiting [8]. Beyond simple attenuation, increasing the diffuse fraction of incoming radiation can enhance within-canopy light penetration and stabilise photosynthetic performance. Emerging evidence indicates that a moderate diffuse fraction of photosynthetically active radiation (PAR; 400–700 nm) can raise canopy-scale carbon gain and yield in crops and crop-like systems by (i) distributing photons more evenly across leaf layers, (ii) reducing localised excitation pressure and excess-energy losses, and (iii) increasing whole-canopy light-use efficiency under high irradiance [9,10]. In controlled-environment horticulture, “smart covers” (diffusive and photoselective) are increasingly viewed as a next-generation approach to tune intensity, spectrum, and angular distribution to plant demand, reinforcing the physiological rationale for field studies that explicitly test radiation regimes [11].

Critically, genotype determines whether radiation moderation translates into higher carbon gain and leaf yield. Recent evaluations report substantial variation among *S. rebaudiana* accessions in leaf biomass, physiological traits, and steviol glycoside profiles under contrasting environments, indicating exploitable functional diversity rather than a single “universal” ideotype [12,13]. In the Colombian Caribbean, radiation has already been identified as a relevant stressor for *S. rebaudiana*, and field evidence suggests that moderating high radiation can improve physiological efficiency metrics even when gross growth rates appear similar [14,15]. In addition, targeted screening of Unicórdoba germplasm has shown genotype-level differentiation in physiological and enzymatic indicators linked to stress tolerance, supporting the use of elite, contrasting materials as a strategic platform for field eco-physiology [16].

Despite these advances, a key knowledge gap remains: few factorial field studies in *S. rebaudiana* have simultaneously resolved the three-way interaction among location (environment/VPD), incident radiation regime, and genotype (genotype × environment × management; G × E × M) using paired gas-exchange and chlorophyll-fluorescence diagnostics, and then linked those mechanisms to leaf yield across multiple tropical environments. Addressing this gap is essential for moving from descriptive agronomy (“yield differs by site”) to deployable inference (“which physiological traits explain site-specific performance, and which genotypes express them reliably”). Here, we leveraged the *S. rebaudiana* germplasm resources of the Universidad de Córdoba [17] and evaluated four contrasting genotypes (L020, L102, L082, and the commercial standard ‘Morita II’) across three Caribbean environments under contrasting incident-radiation conditions.

In this study, we tested the overarching hypothesis that *S. rebaudiana* performance in the lowland tropics is governed by strong genotype × environment contingencies, such that the physiological and agronomic consequences of incident radiation depend on location-specific atmospheric demand (particularly the VPD–temperature context) and on genotype-level capacity to coordinate stomatal regulation with PSII operating efficiency. Specifically, we predicted that moderated incident radiation (lower peak photosynthetic photon flux density (PPFD) and a higher diffuse fraction) would enhance PSII operating efficiency (higher ΦPSII, Fv′/Fm′ and *qP*) and stabilise gas exchange in the hottest/high-VPD environment, whereas direct full sun would favour carbon gain and yield where atmospheric demand is less restrictive; we further anticipated genotype rank changes across locations, consistent with meaningful location × radiation × genotype interactions for both physiological traits and leaf yield. To evaluate these predictions, we deployed four reference genotypes (L020, L102, L082 and the commercial standard ‘Morita II’) across three Caribbean environments under two contrasting radiation regimes and pursued three measurable objectives: (i) to quantify genotype-specific plasticity in leaf gas exchange (*A_N_*, *g_s_*, *E* and C_i_) and light-adapted PSII functionality (ΦPSII, Fv′/Fm′, *qP* and ETR) under contrasting incident radiation; (ii) to test the significance and structure of location × radiation × genotype effects using factorial inference aligned with the split-plot design; and (iii) to explore the association of physiological traits with biomass allocation and leaf yield through complementary correlation analysis and principal component analysis (PCA), thereby providing a trait-based basis for site-tailored light management and genotype deployment in tropical *S. rebaudiana* systems.

## 2. Results

### 2.1. Gas-Exchange Parameters

**Figure 1:**
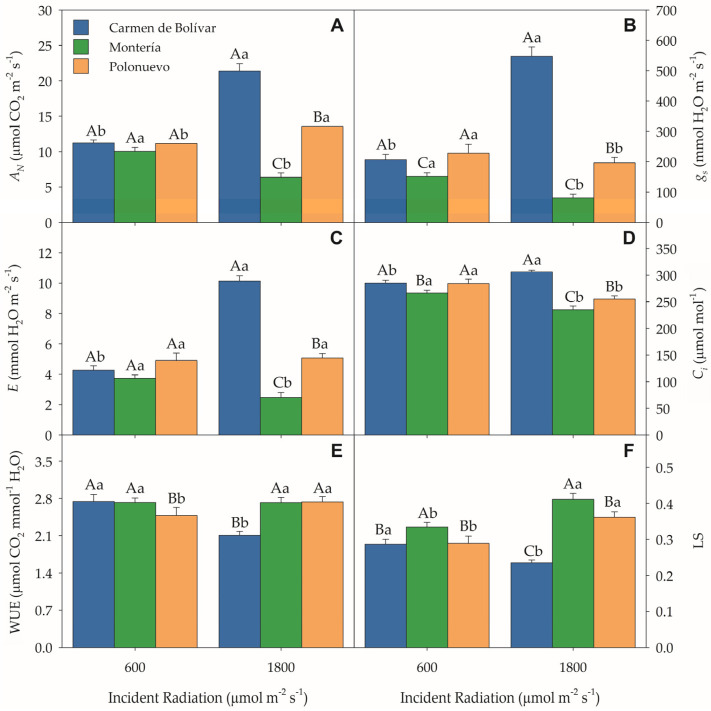
Gas exchange parameters of *Stevia rebaudiana* under contrasting radiation regimes across three Caribbean environments: (**A**) net photosynthetic rate (*A_N_*), (**B**) stomatal conductance (*g_s_*), (**C**) transpiration rate (*E*), (**D**) intercellular CO_2_ concentration (C_i_), (**E**) water-use efficiency (WUE), and (**F**) stomatal limitation (L_s_). Data represent mean ± SE (n = 16). Different uppercase letters indicate significant differences between radiation treatments within each location, whereas lowercase letters indicate differences among locations within each radiation level (Tukey test, *p* ≤ 0.05). Abbreviations: *A_N_*, net photosynthetic rate; *g_s_*, stomatal conductance; *E*, transpiration; C_i_, intercellular CO_2_ concentration; WUE, water-use efficiency; L_s_, stomatal limitation.

A highly significant location × radiation interaction was detected for net photosynthesis (*A_N_*, *p* < 0.0001), stomatal conductance (*g_s_*, *p* < 0.0001), transpiration (E, *p* < 0.0001), intercellular CO_2_ concentration (C_i_, *p* = 0.0031), water-use efficiency (WUE, *p* = 0.0007), and stomatal limitation (L_s_, *p* < 0.0001) (Table S1). Under direct incident radiation (1800 μmol photons m^−2^ s^−1^), *A_N_* increased by 90.2% and 21.5% at El Carmen de Bolívar (21.37 ± 1.06 μmol CO_2_ m^−2^ s^−1^) and Polonuevo (13.55 ± 0.60 μmol CO_2_ m^−2^ s^−1^), respectively, whereas Montería showed a 36.4% reduction relative to plants exposed to moderate radiation (600 μmol photons m^−2^ s^−1^) (Figure 1A). *g_s_* followed a similar pattern to *A_N_*, increasing by 164.7% at El Carmen de Bolívar (547.13 ± 31.19), but decreasing by 46.6% and 13.7% at Montería (80.80 ± 12.64) and Polonuevo (196.59 ± 17.67), respectively (Figure 1B). E increased markedly at El Carmen de Bolívar (+137.5%) and only slightly at Polonuevo (+3.3%) under direct radiation (Figure 1C). In contrast, C_i_ decreased by 11.6% and 10.2% at Montería (235.39 ± 6.65) and Polonuevo (255.32 ± 5.89), respectively, while a modest 7.3% increase was recorded at El Carmen de Bolívar (Figure 1D). WUE did not vary significantly in Montería, but decreased by 23% at El Carmen de Bolívar (2.11 ± 0.07) and increased by 10% at Polonuevo (2.73 ± 0.10) under direct radiation (Figure 1E).

Stomatal limitation (L_s_) reached significantly higher values under direct radiation in Montería (0.41 ± 0.02) and Polonuevo (0.36 ± 0.01), whereas it decreased in El Carmen de Bolívar to 0.23 ± 0.01 (Figure 1F). Because lower L_s_ indicates weaker stomatal limitation (more open stomata) and higher L_s_ indicates stronger stomatal limitation, these results show that direct radiation reduced stomatal limitation at El Carmen de Bolívar but increased stomatal limitation at Montería and Polonuevo, consistent with the site-dependent behaviour observed for *A_N_* and *g_s_*.

### 2.2. Chlorophyll Fluorescence Parameters

**Figure 2:**
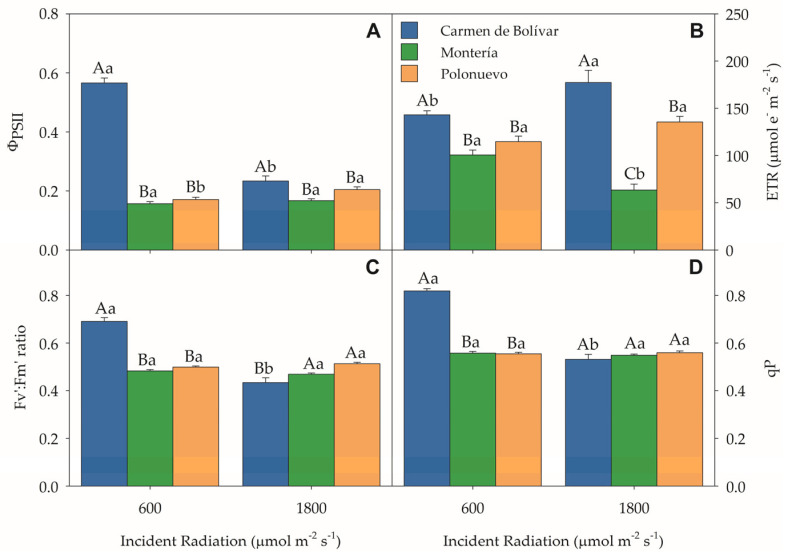
Chlorophyll fluorescence parameters of *Stevia rebaudiana* under two incident radiation levels across three Caribbean locations (Colombia): (**A**) effective quantum yield of PSII (ΦPSII), (**B**) electron transport rate (ETR), (**C**) maximum efficiency of PSII in the light-adapted state (Fv′/Fm′), and (**D**) photochemical quenching coefficient (*qP*). Means followed by uppercase letters indicate significant differences between radiation levels within the same location; lowercase letters indicate significant differences among locations within the same radiation level. Bars represent means ± SE. n = 16. Abbreviations: PSII, photosystem II; ΦPSII, effective quantum yield of PSII; ETR, electron transport rate; Fv′/Fm′, maximum efficiency of PSII in the light-adapted state; *qP*, photochemical quenching coefficient; SE, standard error.

Chlorophyll fluorescence traits were strongly shaped by environment and radiation regime. A highly significant location × radiation interaction was detected for the effective quantum yield of PSII (ΦPSII, *p* < 0.0001), the electron transport rate (ETR, *p* = 0.0019), the maximum efficiency of PSII in the light-adapted state (Fv′/Fm′; *p* < 0.0001), and the photochemical quenching coefficient (*qP*, *p* < 0.0001) (Table S1). Under moderate radiation, plants established in El Carmen de Bolívar showed markedly higher ΦPSII, ETR, and Fv′/Fm′ than those grown in Montería and Polonuevo, with significant increases of 260.51% and 230.99%, 59.22% and 38.48%, and 46.87% and 47.39%, respectively (Figure 2A–D). Under direct radiation, El Carmen de Bolívar also maintained significantly higher ΦPSII (0.234 ± 0.017) and ETR (177.358 ± 12.914 μmol e^−^ m^−2^ s^−1^) than Montería (0.167 ± 0.007; 63.470 ± 6.364 μmol e^−^ m^−2^ s^−1^) and Polonuevo (0.204 ± 0.009; 135.459 ± 6.062 μmol e^−^ m^−2^ s^−1^) (Figure 2A,B). Nevertheless, the remaining fluorescence parameters showed differential site behaviour: Polonuevo exhibited significantly higher Fv′/Fm′ (0.513 ± 0.006) than El Carmen de Bolívar (0.434 ± 0.020), while the latter remained lower than Montería (0.469 ± 0.006); in contrast, *qP* did not differ among locations under direct radiation (Figure 2C,D). Within El Carmen de Bolívar, moderate radiation increased Φ_PSII_, Fv′/Fm′, and *qP* by 141.88%, 59.22%, and 53.76%, respectively, relative to direct radiation, whereas ETR displayed an opposite response (−19.30%) (Figure 2A–D). In Polonuevo, plants under direct radiation showed significantly higher ΦPSII than those under moderate radiation, whereas in Montería, moderate radiation produced a slightly significant 6.36% increase in ΦPSII relative to direct radiation. Collectively, these results indicate that fluorescence performance was highly environment dependent, with El Carmen de Bolívar showing the clearest advantage under moderate radiation across key PSII operating metrics.

**Figure 3:**
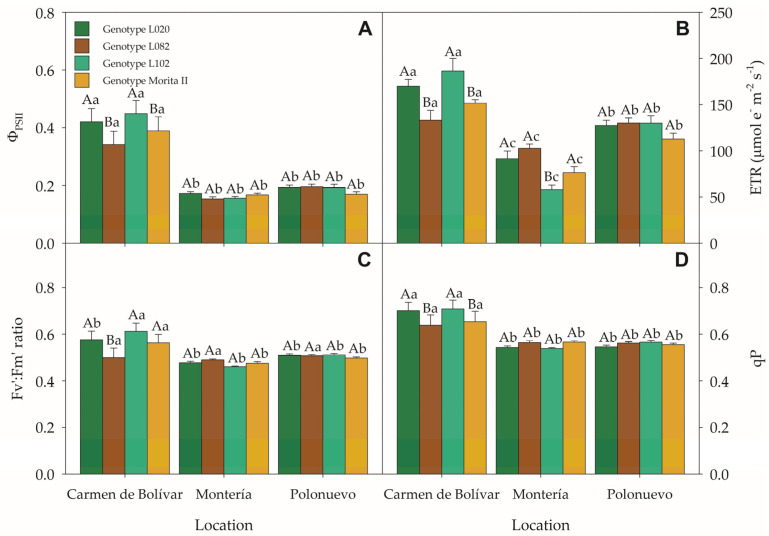
Chlorophyll fluorescence parameters of four *Stevia rebaudiana* genotypes across three Caribbean locations (Colombia): (**A**) effective quantum yield of PSII (ΦPSII), (**B**) electron transport rate (ETR), (**C**) maximum efficiency of PSII in the light-adapted state (Fv′/Fm′), and (**D**) photochemical quenching coefficient (*qP*). Means followed by uppercase letters indicate significant differences among genotypes within the same location; lowercase letters indicate significant differences among locations within the same genotype. Bars represent means ± SE. *n* = 16. Abbreviations: PSII, photosystem II; ΦPSII, effective quantum yield of PSII; ETR, electron transport rate; Fv′/Fm′, maximum efficiency of PSII in the light-adapted state; *qP*, photochemical quenching coefficient; SE, standard error.

A highly significant location × genotype interaction was also observed for ΦPSII (*p* = 0.0025); ETR (*p* < 0.0001); Fv′/Fm′ (*p* < 0.0001); and *qP* (*p* = 0.0007) (Table S1), highlighting genotype-specific photochemical responses across environments (Figure 3A–D). In El Carmen de Bolívar, genotypes L020 and L102 displayed the highest ΦPSII (0.420 ± 0.046; 0.449 ± 0.045) and ETR (169.918 ± 7.326; 186.365 ± 13.667 μmol e^−^ m^−2^ s^−1^), significantly exceeding L082 (0.341 ± 0.046; 133.167 ± 10.555 μmol e^−^ m^−2^ s^−1^) and ‘Morita II’ (0.389 ± 0.048; 151.522 ± 3.726 μmol e^−^ m^−2^ s^−1^), as well as the values recorded for these genotypes in Montería and Polonuevo (Figure 3A,B). Notably, Fv′/Fm′ and *qP* suggested greater photochemical stability in El Carmen de Bolívar (Figure 3C,D). In contrast, Montería and Polonuevo showed overall reductions in ΦPSII and ETR largely irrespective of genotype, indicating lower utilisation of absorbed radiant energy under those site-specific conditions. Overall, genotypic differences were most pronounced in El Carmen de Bolívar, where L020 and L102 maintained a superior and more consistent photochemical performance than the other genotypes.

### 2.3. Biometric Traits

Plant height (PH) and leaf area (LA) were significantly affected by the interactions between location and radiation (PH: *p* < 0.0001; LA: *p* = 0.0051) and between location and genotype (PH: *p* < 0.0001; LA: *p* < 0.0001) in *S. rebaudiana*. Under moderate radiation, plants attained significantly greater PH in Monteria, with increases of 26.68% and 19.32% relative to El Carmen de Bolivar and Polonuevo, respectively. However, under direct radiation, PH decreased in Monteria and Polonuevo by 16.50% and 26.56%, respectively. Across genotypes, L020, L102 and ‘Morita II’ significantly outperformed L082, with L102 and ‘Morita II’ recording the highest PH values in Monteria (71.13 ± 1.70 and 71.12 ± 1.63 cm). In contrast, L082 consistently showed the lowest PH across all locations, suggesting limited phenotypic plasticity and a markedly negative response to contrasting environments.

Leaf area exhibited pronounced location-dependent responses. In Monteria, LA under moderate radiation increased significantly by 225.20% and 21.24% compared with El Carmen de Bolivar and Polonuevo, respectively. Conversely, under direct radiation, plants established in Polonuevo experienced a 34.27% reduction in LA, indicating constrained leaf expansion under excess incident radiation at that site. In the genotype analysis, L020, L102 and ‘Morita II’ exhibited significantly higher LA than L082, which consistently presented the lowest values across all locations. The highest LA was observed in Monteria for L020 (2043.60 ± 86.60 cm^2^), exceeding Polonuevo (1204.82 ± 113.29 cm^2^) and El Carmen de Bolivar (654.59 ± 39.16 cm^2^) by 69.6% and 212.2%, respectively. Both L102 and ‘Morita II’ also reached their highest LA values in Monteria compared with the other environments. Overall, these patterns indicate that direct radiation limited leaf expansion in Polonuevo, but the magnitude of the response depended on both location and genotype, with L082 consistently showing reduced adaptive capacity. These location- and genotype-dependent patterns for PH and LA are summarised in Table 1.

A significant location × radiation × genotype interaction was detected for leaf area index (LAI) (*p* = 0.0030), number of leaves (NL) (*p* = 0.0069), root dry weight (RDW) (*p* = 0.0058), stem dry weight (SDW) (*p* = 0.0015), leaf dry weight (LDW) (*p* = 0.0174) and total dry weight (TDW) (*p* = 0.0092) in *S. rebaudiana* (Table S1). As shown in Table 2, genotypes L020, L102 and ‘Morita II’ displayed higher LAI, NL, RDW, SDW, LDW and TDW in almost all locations, largely irrespective of radiation level. In contrast, L082 exhibited the lowest values across all traits, with reductions in TDW and LAI ranging from 70 to 85% relative to L020 and L102, confirming its consistently poor performance across environments. The magnitude and direction of these three-way responses across canopy and biomass traits are detailed in Table 2.

Accordingly, in El Carmen de Bolivar and Monteria, direct radiation significantly increased dry-matter accumulation (RDW, SDW, LDW and TDW) in most genotypes, with L020 and L102 standing out as the most responsive materials. In particular, LDW increased significantly by 140% (L020) and 133% (L102) compared with plants under moderate radiation. A similar pattern was observed for TDW, with significant mean increases of up to 80% and 70% for L102 and L020, respectively. By contrast, in Polonuevo, exposure to direct radiation resulted in reductions of 35% (LAI) and 25% (TDW), suggesting that excessive radiation imposed a limiting effect on canopy development and total biomass production at this site. The most notable contrasts were observed among locations. Monteria emerged as the most favourable location for the expression of LAI and NL. Under direct radiation, the LAI of L020 and L102 in Monteria exceeded those in El Carmen de Bolivar by 242.9% and 657%, and those in Polonuevo by 242% and 498.5%, respectively. Likewise, for both genotypes, NL increased by 418.6% and 460.3% relative to El Carmen de Bolivar, and by 373.7% and 394.4% relative to Polonuevo. Nevertheless, dry-matter accumulation (RDW, SDW, LDW and TDW) was more determinant in El Carmen de Bolivar than in the other locations. In El Carmen de Bolivar, under direct radiation, L020 and L102 recorded higher RDW, SDW and LDW than in Monteria and Polonuevo; similarly, under moderate radiation, RDW, SDW and LDW also exceeded the values observed in Monteria and Polonuevo. For TDW, under direct radiation, L020 increased significantly in El Carmen de Bolivar by 34.78% (vs. Monteria) and 325.24% (vs. Polonuevo), while L102 increased by 28.45% (vs. Monteria) and 320.78% (vs. Polonuevo). Under moderate radiation, TDW increased by 71.37% and 143.49% for L020, and by 38.11% and 261.10% for L102, relative to Monteria and Polonuevo, respectively. Collectively, these results suggest that Monteria enhanced canopy traits (LAI and NL), whereas El Carmen de Bolivar favoured total biomass accumulation for L020 and L102 regardless of radiation level, consolidating these genotypes as promising materials. In contrast, L082 showed a consistently deficient response across all traits, irrespective of location and radiation regime.

### 2.4. Leaf Yield and Harvest Index

**Figure 4:**
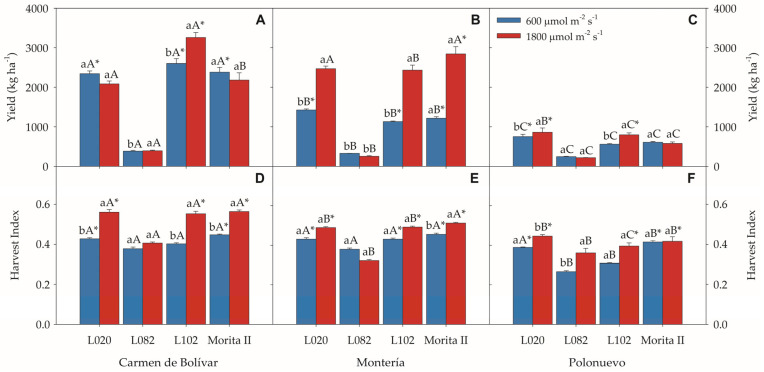
Leaf yield and harvest index of *Stevia rebaudiana* under two incident radiation regimes across three Caribbean locations (Colombia): (**A**–**C**) leaf yield (Y) and (**D**–**F**) harvest index (HI). Bars represent means ± SE. Different uppercase letters indicate significant differences between radiation treatments within each location, whereas lowercase letters indicate significant differences among locations within each radiation level (Tukey test, *p* ≤ 0.05). Abbreviations: Y, leaf yield; HI, harvest index; SE, standard error. (*) indicate significant differences among locations within the same radiation level and genotype (Tukey test, *p* ≤ 0.05).

A significant location × radiation × genotype interaction was detected for leaf yield (*p* = 0.0176) and harvest index (HI) (*p* = 0.0345) in *S. rebaudiana* (Table S1). Overall leaf yield was highest in El Carmen de Bolívar (1951.46 ± 182.03 kg ha^−1^), followed by Montería (1510.94 ± 173.75 kg ha^−1^), and lowest in Polonuevo (576.31 ± 42.36 kg ha^−1^) (Figure 4A–C). In El Carmen de Bolívar, genotype L102 achieved the highest leaf yield under direct radiation (3256.25 ± 126.39 kg ha^−1^) relative to plants exposed to moderate radiation; L082 showed only a 2.4% increase, whereas L020 and ‘Morita II’ exhibited slight decreases of 11% and 8.4%, respectively, under higher radiation (Figure 4A). In Montería, direct radiation increased leaf yield in L020, L102, and ‘Morita II’ by 73.1%, 115.3%, and 134.4%, respectively (2467.34 ± 68.71; 2430.65 ± 129.94; 2842.74 ± 184.32 kg ha^−1^), whereas L082 reduced leaf yield by 22.9% (Figure 4B). In Polonuevo, direct radiation had a positive effect on yield for L020 and L102 (862.50 ± 105.31 and 795.52 ± 51.09 kg ha^−1^), although yields remained comparatively low relative to the other locations (Figure 4C).

Harvest index, reflecting the efficiency of biomass allocation to the commercial organ, also exhibited strong environmental contingency (Figure 4D–F). In El Carmen de Bolívar, harvest index reached significantly higher values under direct radiation for L020 (0.56 ± 0.01), L102 (0.55 ± 0.01), and ‘Morita II’ (0.56 ± 0.01) compared with plants under moderate radiation (Figure 4D). In Montería, harvest index showed no significant differences between radiation levels for L020, L102, and L082, whereas ‘Morita II’ stood out with a value of 0.51 ± 0.001 (Figure 4E). In Polonuevo, harvest index of L082 was lower under moderate radiation (0.36 ± 0.02) than under direct radiation (Figure 4F). Overall, these results indicate that yield formation and biomass partitioning in *S. rebaudiana* are jointly determined by location, incident radiation, and genotype, with L102 and ‘Morita II’ showing consistently competitive yield and harvest-index values across environments. Any inference on physiological stability or breeding relevance is therefore integrated with the underlying gas-exchange and fluorescence responses in the Discussion.

### 2.5. Pearson Correlation Analysis

**Figure 5:**
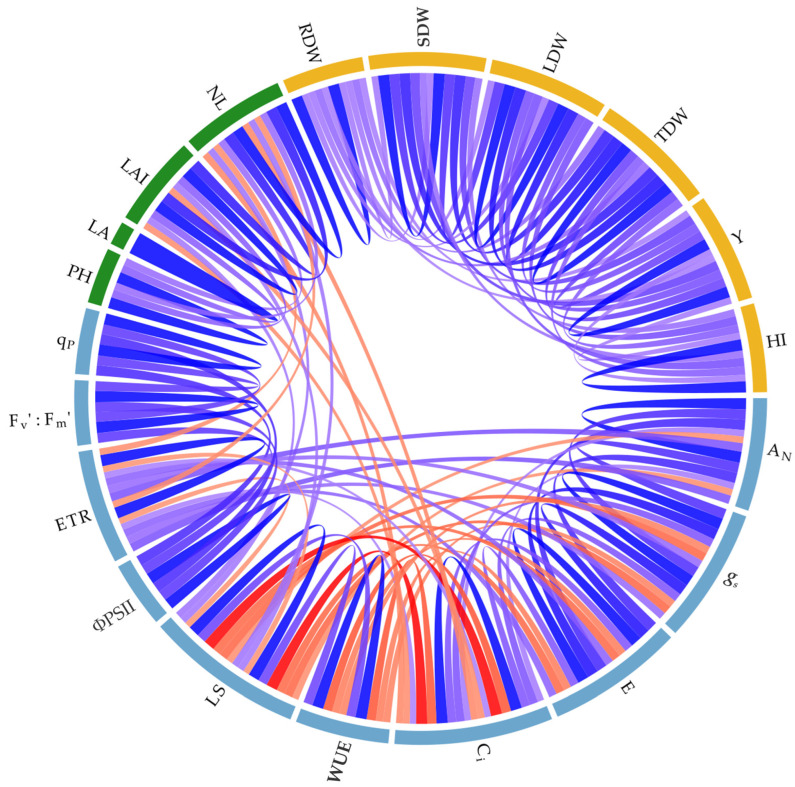
Chord diagram of Pearson correlations among physiological, morphometric, and biomass/yield traits in *S. rebaudiana* evaluated across three Caribbean locations (Colombia) under contrasting incident radiation. Red indicates negative correlations (−1), whereas blue indicates positive correlations (+1). Variables are grouped by functional category: light blue, gas exchange and chlorophyll fluorescence traits; green, morphometric traits; and gold, biomass and yield traits. Abbreviations: *A_N_*, net photosynthetic rate; *g_s_*, stomatal conductance; *E*, transpiration; C_i_, intercellular CO_2_ concentration; WUE, water-use efficiency; ΦPSII, effective quantum yield of PSII; ETR, electron transport rate; Fv′/Fm′, maximum efficiency of PSII in the light-adapted state; *qP*, photochemical quenching coefficient; L_s_, stomatal limitation; PH, plant height; LA, leaf area; LAI, leaf area index; NL, number of leaves; RDW, root dry weight; SDW, stem dry weight; LDW, leaf dry weight; TDW, total dry weight; Y, leaf yield; HI, harvest index.

Pearson correlation analysis was used as a complementary exploratory approach to summarise associations among gas exchange, chlorophyll fluorescence, morphometric traits, and biomass/yield variables in *S. rebaudiana* (Figure 5; Table S2). As expected, net photosynthesis (*A_N_*) was positively associated with stomatal conductance (*g_s_*), transpiration (*E*), and the effective quantum yield of PSII (ΦPSII), reflecting coordinated relationships among carbon assimilation, stomatal behaviour, and photochemical performance under field conditions. Likewise, morphometric traits tended to be positively associated with biomass components and leaf yield (Y).

In contrast, water-use efficiency (WUE) showed negative associations with several gas-exchange variables, consistent with the covariation among carbon gain, transpirational flux, and internal CO_2_ diffusion across contrasting environments. Because many of these relationships are well established in plant ecophysiology, the correlation analysis is presented here only as a complementary overview of trait coherence, while the main interpretation of treatment effects is based on radiation- and location-dependent responses.

### 2.6. Multivariate Analysis (Principal Component Analysis, PCA)

**Figure 6:**
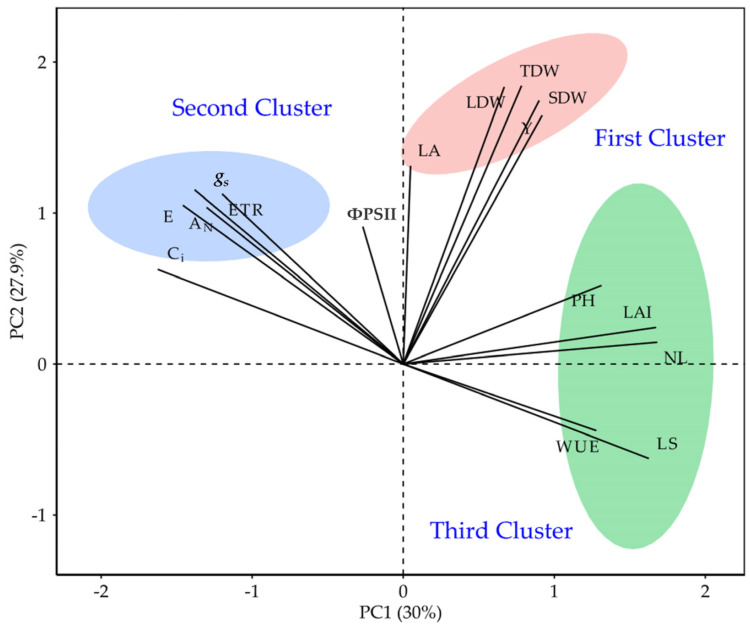
PCA biplot of physiological (gas exchange and chlorophyll fluorescence), morphometric, biomass, and yield-related traits in *Stevia rebaudiana*. The first two principal components explained 61.9% of the total variance (PC1 = 30.0%, PC2 = 27.9%). Vectors indicate trait loadings and points represent observations across locations, radiation regimes, and genotypes. Abbreviations: PCA, principal component analysis; *A_N_*, net photosynthetic rate; *g_s_*, stomatal conductance; *E*, transpiration; C_i_, intercellular CO_2_ concentration; WUE, water-use efficiency; L_s_, stomatal limitation; ΦPSII, effective quantum yield of PSII; ETR, electron transport rate; Fv′/Fm′, maximum efficiency of PSII in the light-adapted state; *qP*, photochemical quenching coefficient; PH, plant height; LA, leaf area; LAI, leaf area index; NL, number of leaves; RDW, root dry weight; SDW, stem dry weight; LDW, leaf dry weight; TDW, total dry weight; Y, leaf yield.

**Figure 7:**
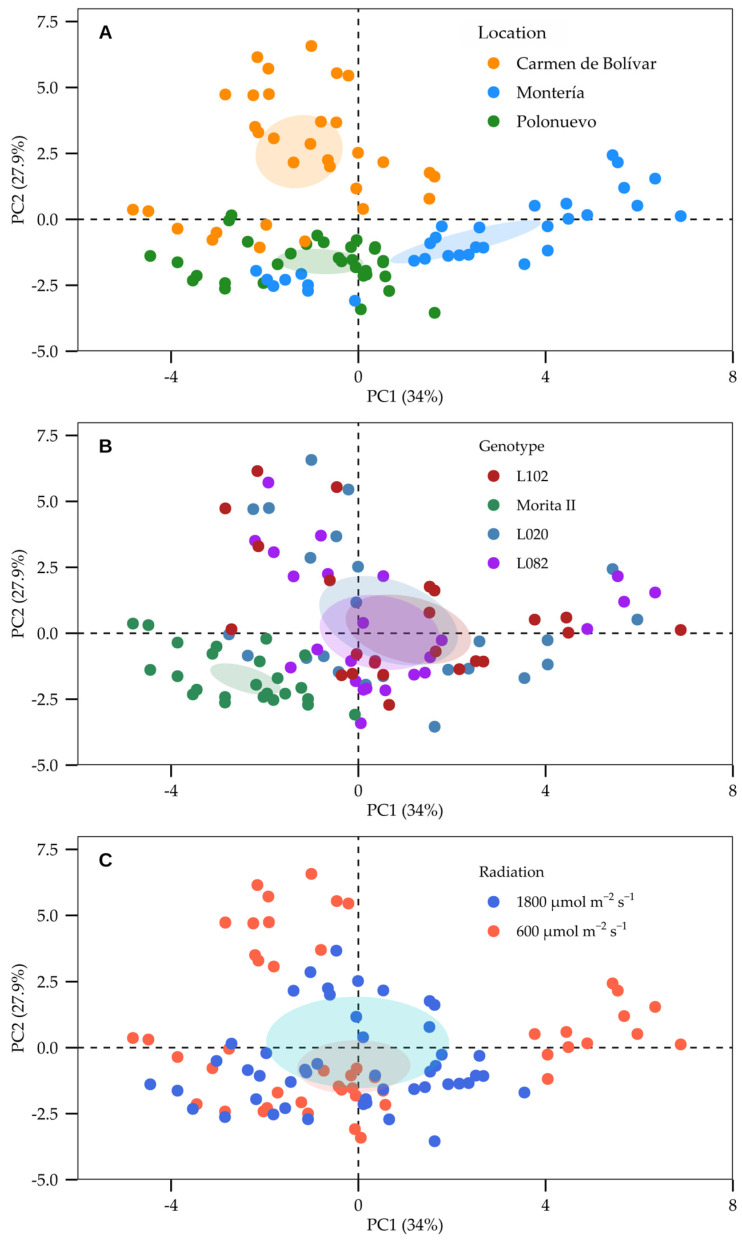
PCA score plots showing the distribution of observations according to (**A**) location, (**B**) genotype, and (**C**) incident radiation regime in *Stevia rebaudiana* evaluated in the Caribbean region of Colombia. Shaded areas were included to visually highlight the main clusters associated with the corresponding groups in each panel.

Principal component analysis (PCA) integrated gas-exchange, chlorophyll fluorescence, morphometric traits, biomass components, and yield-related variables to describe the multivariate structure of phenotypic variation across locations, radiation regimes, and genotypes (Figure 6 and Figure 7). The first two principal components explained 61.9% of the total variance (PC1 = 30.0%; PC2 = 27.9%), providing a compact representation of the dominant axes of covariation among traits (Figure 6). PC1 was mainly driven by NL (11.31%), LAI (11.22%), L_s_ (10.57%), and C_i_ (10.57%), indicating that this axis captured a combined gradient of canopy development and CO_2_ diffusion/stomatal limitation traits (Table S3). In parallel, *A_N_*, *g_s_*, *E*, ETR and ΦPSII clustered in the same multivariate space, reflecting the close coupling between carbon assimilation and PSII photochemical performance, whereas PC2 was dominated by biomass and productivity variables, with the highest contributions from TDW (13.58%), LDW (13.47%), SDW (12.17%), and leaf yield (Y; 10.84%) (Figure 6; Table S3). Together, these loadings indicate that the primary multivariate separation of observations involved coordinated variation in canopy/CO_2_ diffusion traits (PC1) and dry-matter accumulation and yield formation (PC2).

Scores and trait vectors further demonstrated that location was the main source of multivariate differentiation (Figure 7A). Montería aligned more closely with morphometric traits associated with canopy size (NL and LA), whereas El Carmen de Bolívar showed stronger association with gas-exchange and photochemical variables (*A_N_*, *g_s_*, *E*, C_i_, ETR and ΦPSII), and Polonuevo exhibited a distinct positioning related to growth and water-use traits (PH, NL, LAI and WUE) (Figure 7A). Genotype ordination suggested that L020 and L102 were more strongly associated with dry-matter accumulation traits (RDW, SDW, LDW and TDW), while ‘Morita II’ aligned more closely with photochemical variables, and L082 occupied an intermediate region consistent with its comparatively lower expression of morphometric and biomass traits across environments (Figure 7B). Radiation regime also contributed to multivariate shifts: direct radiation clustered more closely with growth and dry-mass variables, whereas moderate radiation showed greater association with traits linked to photosynthetic adjustment and stomatal regulation, indicating meaningful physiological plasticity under contrasting incident radiation (Figure 7C).

## 3. Discussion

### 3.1. Environment-Dependent Photosynthetic Regulation Under Contrasting Incident Radiation

This study provides clear evidence that the physiological response of *Stevia rebaudiana* to incident radiation is strongly environment-dependent, revealing a marked G × E component in carbon–water relations. Across locations, the shift from moderate (≈600 µmol photons m^−2^ s^−1^) to high radiation (≈1800 µmol photons m^−2^ s^−1^) elicited contrasting patterns of net photosynthesis (A_n_), stomatal conductance (*g_s_*), intercellular CO_2_ concentration (C_i_), and stomatal limitation (L_s_). In Carmen de Bolívar, high radiation increased A_n_ while also increasing *g_s_* and slightly raising C_i_, indicating that enhanced CO_2_ diffusion supported assimilation under high irradiance. In contrast, Montería and Polonuevo displayed a reduction in *g_s_* and C_i_ under high radiation, accompanied by a stronger stomatal limitation signal (higher L_s_), consistent with an environment-triggered stomatal constraint on CO_2_ supply that ultimately reduced A_n_, particularly in Montería.

The climatic differences among the experimental locations provide an important context for interpreting these responses. In tropical environments, incident radiation often co-occurs with variations in air temperature and atmospheric demand, which can substantially influence stomatal behaviour and photosynthetic regulation. Higher evaporative demand associated with elevated temperature and vapor pressure deficit (VPD) can promote stomatal restriction, thereby limiting CO_2_ diffusion and altering the balance between photochemical energy use and carbon assimilation. Consequently, the contrasting physiological responses observed among locations likely reflect not only differences in incident radiation but also the interaction between radiation load and site-specific atmospheric conditions. Relative humidity, together with temperature, shapes VPD and may therefore contribute to the observed site-dependent stomatal behaviour. Precipitation patterns may further modulate soil water availability and, in turn, interact with atmospheric demand to shape stomatal regulation and carbon gain across sites.

These divergent responses are ecophysiologically plausible in tropical field settings where “radiation” is not an isolated driver: higher irradiance typically co-varies with leaf-to-air vapour pressure deficit, boundary-layer conditions, and canopy/soil thermal loads. Although these microclimatic covariates were not quantified here (see Section 3.8), the observed combination of decreased *g_s_* and reduced C_i_ under high radiation in Montería and Polonuevo is consistent with a stomatal-regulated limitation to carbon gain under potentially higher atmospheric demand and heat load—conditions commonly reported to constrain assimilation even when light is not limiting. From a management viewpoint, these results imply that “more light” is not universally beneficial for *S. rebaudiana* photosynthesis; rather, the net outcome depends on whether the local environment allows stomatal openness and biochemical use of absorbed energy to be sustained [18,19,20]. The sensitivity of leaf yield and physiological performance to site conditions aligns with broader evidence that *S. rebaudiana* productivity and functional traits vary substantially along environmental gradients, reinforcing the need for site-specific recommendations [21,22,23].

### 3.2. Photochemistry Under Field Radiation: Balancing PSII Efficiency and Electron Transport

Light-adapted chlorophyll fluorescence further clarified how each location modulated the use of absorbed light energy [24]. Carmen de Bolívar exhibited consistently higher PSII operating efficiency (ΦPSII), photochemical quenching (*qP*), and electron transport rate (ETR) relative to Montería and Polonuevo, particularly under moderate radiation, indicating that a larger fraction of absorbed energy remained available for photochemistry rather than being diverted to non-photochemical pathways or dissipative losses [25,26]. Importantly, within Carmen de Bolívar the moderate-radiation regime tended to enhance photochemical metrics compared with full sun, suggesting that even where AN increased under high irradiance, PSII efficiency could still decline—an expected signature of regulatory downshifts or emerging excitation pressure at high photon flux [27,28,29].

Because leaf optical properties were not independently measured, ETR values should be interpreted cautiously as relative fluorescence-derived estimates rather than absolute cross-environment measures of electron flux.

In Montería, the reduction of AN under full sun, together with lower ΦPSII/ETR and stronger stomatal limitation, supports a coupled interpretation: when stomatal closure reduces CO_2_ availability, electron transport can become less effectively used for carbon fixation, increasing the need for protective energy dissipation and potentially elevating photoinhibitory risk if sustained [5,30,31]. These interpretations are consistent with recent evidence showing that UV and high-energy radiation components can compromise *S. rebaudiana* growth and physiological balance depending on exposure regime, while controlled light environments can improve performance by optimising the relationship between photochemistry and downstream carbon metabolism [32,33]. Likewise, controlled-spectrum light studies highlight that plant responses depend not only on intensity but also on the capacity to process excitation energy through photochemical and metabolic sinks [34,35].

Notably, the present fluorescence dataset did not include non-photochemical quenching (NPQ) indices, which prevents a complete quantification of the photoprotection–photochemistry trade-off (see Section 3.8). Adding NPQ (and/or qN) would allow a stronger mechanistic separation between (i) reduced ΦPSII due to protective down-regulation versus (ii) genuine photodamage or persistent limitations in carbon sinks.

### 3.3. From Instantaneous Physiology to Canopy Development and Biomass Partitioning

The growth outcomes demonstrate that physiological performance translated into distinct canopy architectures and biomass allocation strategies across environments [36,37]. Montería produced the largest plants (height, leaf number, leaf area, and leaf area index), whereas Carmen de Bolívar accumulated the highest total dry biomass, implying that greater canopy expansion did not necessarily maximise dry matter accumulation at the whole-plant level [38,39,40]. This decoupling can occur when rapid leaf-area deployment increases respiratory or hydraulic costs, or when environmental constraints reduce the efficiency of converting light capture into structural biomass [39,41,42].

Genotypic differences were also pronounced and biologically interpretable. L082 showed consistently weak performance across multiple traits (leaf area, dry weights, and yield), suggesting limited plasticity and/or lower functional capacity under the tested tropical conditions [43,44,45]. Conversely, L020 expressed strong total biomass and total dry weight, while Morita II exhibited comparatively stable dry biomass across locations—an agronomically relevant feature for production reliability [44,46]. The fact that Carmen de Bolívar combined high A_n_ (under full sun) with superior dry matter accumulation supports the idea that, at least for some genotypes and environments, *S. rebaudiana* can translate high irradiance into productive carbon gain when stomatal openness and photochemical function remain sufficiently preserved [4,30,47]. This is compatible with field evidence that *S. rebaudiana* yield and quality traits can be strongly shaped by light supply and local climate, reinforcing that management decisions on light environment should be environment- and genotype-specific rather than universal [15,21,22].

### 3.4. Yield Formation and Genotype-Specific Radiation Responses Across Locations

Leaf yield patterns provided the clearest agronomic expression of G × E. Carmen de Bolívar achieved the highest mean leaf yield per plant, followed by Montería and then Polonuevo. Radiation effects were not uniform: yield increased under full sun in Carmen de Bolívar and Montería, but in Polonuevo yield was higher under moderate radiation. This cross-over pattern is a classic manifestation of G × E in productive traits and indicates that the “optimal” radiation regime for biomass allocation to harvestable leaves depended strongly on the local environmental context. Indeed, these location-dependent yield responses were likely shaped by the broader climatic and edaphic framework of each site. During the experimental period, Montería combined the highest mean temperature, relative humidity, and accumulated rainfall, whereas El Carmen de Bolívar showed slightly lower humidity and intermediate rainfall, and Polonuevo recorded the lowest precipitation despite a slightly lower mean temperature during the experimental period. In addition, the sites differed in edaphic attributes, including organic carbon, phosphorus, exchangeable bases, and micronutrient availability, indicating that the location effect cannot be interpreted as a radiative context alone. Under this combined climatic and edaphic framework, the strong productive response observed in El Carmen de Bolívar under full sun may reflect a more favourable balance between radiation supply, atmospheric demand, and soil resource availability, whereas the lower performance in Polonuevo suggests that reduced rainfall and less favourable local conditions constrained the translation of incident radiation into harvestable leaf biomass. Thus, the location effect detected here should be understood as an integrated environmental signal emerging from the interaction between radiation regime and site-specific climatic and soil conditions.

These location-dependent yield responses were likely shaped by the broader environmental context of each site. During the experimental period, Montería combined the highest mean temperature, relative humidity, and accumulated rainfall, whereas El Carmen de Bolívar showed slightly lower humidity and intermediate rainfall, and Polonuevo recorded the lowest precipitation despite a slightly lower mean temperature according to the recorded meteorological conditions. In addition, the sites differed in edaphic attributes, including organic carbon, phosphorus, exchangeable bases, and micronutrient availability, indicating that the location effect cannot be interpreted as a radiative context alone. Under this combined climatic and edaphic framework, the strong productive response observed in El Carmen de Bolívar under full sun may reflect a more favourable balance between radiation supply, atmospheric demand, and soil resource availability, whereas the lower performance in Polonuevo suggests that reduced rainfall and less favourable local conditions constrained the translation of incident radiation into harvestable leaf biomass. Thus, the location effect detected here should be understood as an integrated environmental signal emerging from the interaction between radiation regime and site-specific climatic and soil conditions.

At the genotype level, L102 emerged as a high-performing line in Carmen de Bolívar under full sun (peak leaf yield within the trial), indicating that certain segregating lines can capitalise on high radiation when the site permits favourable gas exchange and sufficient photochemical functioning. Morita II ranked among the top genotypes across environments and displayed competitive yield, particularly under full sun in Carmen de Bolívar, supporting its agronomic relevance and relative stability. In contrast, the uniformly low yield of L082 (including under full sun in Montería) suggests poor adaptation and limited value for further selection unless specific traits justify its retention.

These results reinforce the breeding logic that ideotype selection in *S. rebaudiana* should explicitly incorporate environmental stratification. Multi-location evidence from *S. rebaudiana* has shown that genotype rankings and stability can shift across environments, and that AMMI/GGE approaches can help identify stable and high-yielding genotypes under varying conditions [48]. In this context, the present dataset supports a targeted recommendation: L102 is promising for high-radiation sites resembling Carmen de Bolívar, while Polonuevo-like environments may benefit from moderated radiation as a strategy to sustain leaf yield.

### 3.5. Trait Covariation Reveals Coordinated Carbon–Water–Photochemistry Controls

Correlation analysis strengthened the causal coherence between physiological regulation and growth/yield outcomes [49]. Positive associations among *A_N_*, *g_s_*, transpiration (E), ΦPSII, and ETR indicate that, across genotypes and environments, higher carbon gain tended to occur when stomata remained more open [50] and PSII electron transport remained effectively engaged—consistent with an integrated “high-throughput” photosynthetic phenotype. Conversely, the strong negative relationship between water-use efficiency (WUE) [51] and both *g_s_* and E reflects an expected trade-off [50]: higher stomatal openness supports carbon gain but increases water loss, whereas higher WUE often emerges under tighter stomatal control [51]. The negative association between WUE and *A_N_* observed here suggests that, in these environments, high WUE was frequently achieved via stomatal restriction rather than biochemical gains in carbon assimilation per unit water [50].

At the canopy level, positive relationships between leaf area index and leaf yield support the idea that canopy development remains a key proximal driver of harvestable biomass. However, the location-dependent nature of these relationships (e.g., Montería showing strong canopy expansion but not the highest total dry biomass) emphasises that structural traits alone are insufficient: their productive value depends on whether the environment allows sustained physiological efficiency under prevailing radiation and microclimate.

### 3.6. Multivariate Structure (PCA) Supports an Interpretable Physiological–Agronomic Axis of Differentiation

Principal component analysis provided an integrative view of how traits jointly structured variation across genotypes, radiation regimes, and locations. The first two principal components explained a substantial share of overall variation (≈62%), supporting the existence of a reduced-dimensional phenotype space that remains physiologically interpretable. PC1 was primarily driven by canopy and gas-exchange constraint variables (leaf number, LAI, stomatal limitation, and C_i_), indicating that a major axis of differentiation captured variation in canopy deployment together with stomatal–diffusive control. PC2 was mainly associated with biomass and productivity traits (total, leaf, and stem dry weights and leaf yield), reflecting a second axis linked to realised growth and harvestable output.

The clustering of *A_N_* with *g_s_*, ETR, and ΦPSII in the multivariate space supports the mechanistic interpretation that, across environments, assimilation was tightly coupled to both stomatal conductance and photochemical capacity. Meanwhile, the separation of yield/biomass variables along PC2 indicates that productivity emerged as a partially distinct outcome integrating, but not reducible to, instantaneous physiology—consistent with the observed site-specific translation of physiological performance into biomass partitioning and yield [36,52,53]. Collectively, these PCA patterns reinforce the manuscript’s central narrative: G × E in *S. rebaudiana* is not only statistically detectable but also physiologically meaningful, reflecting coordinated variation in light capture, diffusive limitations, photochemistry, and biomass allocation [36,54,55].

### 3.7. Agronomic Implications: Toward Environment-Specific Light Management and Genotype Recommendation

From a practical standpoint, the data indicate that light management for *S. rebaudiana* in tropical field systems should be treated as an environment-specific lever rather than a fixed prescription. Carmen de Bolívar supported high *A_N_* and high yield under full sun for key genotypes (notably L102), suggesting that high radiation can be agronomically beneficial where stomatal limitation is not strongly induced and photochemistry remains sufficiently robust. In Polonuevo, however, moderate radiation improved yield relative to full sun, implying that partial radiation attenuation (e.g., shading strategies) may stabilise functional performance and leaf productivity under that environment.

For breeding and selection, the results support advancing genotypes with (i) high yield potential under high radiation in favourable sites (e.g., L102 in Carmen de Bolívar-like environments) and (ii) resilience or performance under moderated radiation in more constraining sites (Polonuevo-like). Integrating multi-environment information is essential to avoid overgeneralising from single-location trials and to ensure that “high-performing” genotypes are recommended within the environmental domain where their physiology translates reliably into yield [48].

### 3.8. Limitations and Future Directions Integrated Toward the Conclusions

Several targeted limitations—framed as opportunities—can further elevate the predictive and mechanistic strength of this work. First, microclimate was not quantified during gas-exchange measurements (e.g., vapour pressure deficit, leaf temperature, soil moisture), even though these variables are critical for interpreting the Montería vs. Carmen de Bolívar divergence in stomatal limitation and carbon gain. Adding continuous microclimate covariates would allow the radiation effect to be partitioned into “photon-driven” versus “atmospheric/thermal-demand-driven” components, strengthening causal interpretation. Second, fluorescence measurements did not include NPQ (or qN), preventing a complete quantification of the photoprotection–photochemistry balance; adding NPQ would refine the mechanistic explanation of how PSII regulation differs across environments and radiation regimes [32,33]. Third, the study represents a single evaluation window; repeating the trial across seasons or years would materially increase inference on G × E stability and would allow robust separation of transient versus repeatable environment effects. Fourth, steviol glycoside (SG) concentration and composition were not quantified in this experiment, so we cannot directly test how location, genotype, and incident radiation shape sweetener quality. Future multi-environment trials should integrate targeted SG profiling (e.g., HPLC/UPLC quantification of stevioside and rebaudioside A) and report both SG concentration (mg g^−1^ DW) and SG yield (g plant^−1^), linking quality with the physiological and yield plasticity documented here.

Finally, the G × E interpretation could be extended from “descriptive” to “predictive” by integrating stability and envirotyping frameworks. Complementing the current approach with AMMI/GGE stability modelling—as applied in *S. rebaudiana* yield stability studies—would strengthen genotype recommendation under multi-environment deployment [48]. In addition, modern enviromics/envirotyping strategies can formalise environmental characterisation and enable prediction across untested site–year combinations by leveraging structured environmental covariates [56] and by identifying environment types and adaptation zones using machine-learning–assisted classification in multi-environment data [57]. These additions would naturally connect with the conclusions by reinforcing that the observed location × radiation × genotype interactions are not merely statistical artefacts, but actionable signals for (i) environment-tailored management and (ii) ideotype/line advancement strategies for tropical *S. rebaudiana* systems.

## 4. Materials and Methods

### 4.1. Plant Material, Field Establishment, and Environmental Conditions

Four *Stevia rebaudiana* genotypes (L020, L082, L102 and ‘Morita II’) were selected from the *S. rebaudiana* germplasm bank of the Universidad de Córdoba based on elite agronomic attributes, including high water-use efficiency, high biomass production, and a robust antioxidant system [16]. Mother plants were maintained for three months in a Type-I biospace (1000 m^2^) under semi-controlled conditions (28 °C, 85% relative humidity, and a photosynthetic photon flux density (PPFD) of 600 μmol photons m^−2^ s^−1^). Clonal multiplication was performed using stem cuttings rooted for 20 days in germination trays filled with coconut peat substrate to promote root development and the formation of two leaves. Rooted cuttings were transplanted into polyethylene bags containing approximately 1.9 L of substrate (17 cm height × 12 cm diameter) filled with soil from the Montería region (pH 6.08, organic matter 1.64%, cation exchange capacity 18.8 cmol (+) kg^−1^, and silty clay loam texture). After a 30-day acclimation period, seedlings reached an average height of 20 cm and developed four functional leaves, which were considered suitable for field establishment.

**Figure 8:**
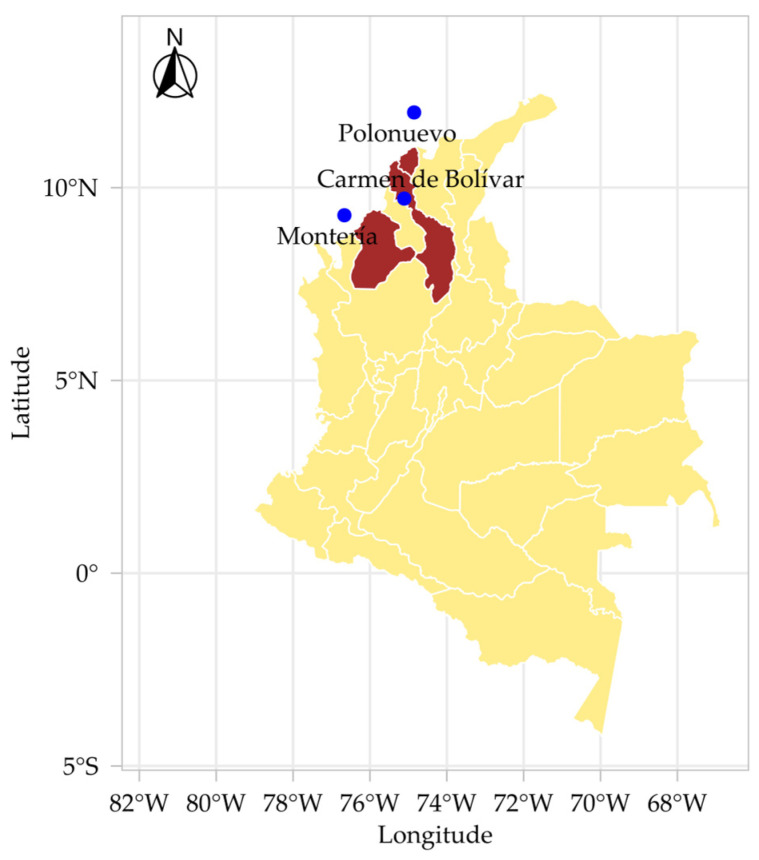
Geographic location of the three experimental sites in the Caribbean region of Colombia: El Carmen de Bolívar (Bolívar), Montería (Córdoba), and Polonuevo (Atlántico). The map indicates the spatial distribution of locations used for the multi-environment evaluation of *Stevia rebaudiana* under contrasting incident-radiation conditions.

The experiment was conducted simultaneously from 28 June to 28 September 2024 at three representative Caribbean sites in Colombia: (i) Carmen de Bolívar, Bolívar (9°42′56″ N, 75°6′27″ W; 155 m a.s.l.), at the Corporación Colombiana de Investigación Agropecuaria (Agrosavia), Turipaná station; (ii) Montería, Córdoba (8°75′64″ N, 75°88′91″ W; 18 m a.s.l.), at the experimental campuses of the Faculty of Agricultural Sciences, Universidad de Córdoba; and (iii) Polonuevo, Atlántico (10°76′70″ N, 74°51′0″ W; 80 m a.s.l.), at Finca El Líbano (Figure 8). Soil physicochemical properties for each site are presented in Table 3.

Seedlings were transplanted at a uniform spacing of 0.4 m × 0.4 m (62,500 plants ha^−1^). Plots consisted of six rows, each 4 m in length, and were established under two incident-radiation environments representing contrasting levels of incident PPFD: (i) moderate radiation using black high-density polyethylene shade nets with UV additive (60% transmittance) and (ii) direct radiation (full exposure; 100% transmittance). At each location, a sampled population of 1280 plants was established within an experimental area of 204.8 m^2^.

Crop management (irrigation and fertilisation). To minimise potential confounding effects of water or nutrient limitations on photosynthesis and leaf yield, all experimental plots received uniform agronomic management within each location. Supplemental irrigation was applied by micro-sprinklers to maintain soil moisture near optimal conditions throughout the experimental period. Irrigation scheduling was based on crop evapotranspiration (ETc), estimated from reference evapotranspiration (ETo) and a crop coefficient (Kc) of 1.2 using the relationship ETc = Kc × ETo.

Soil volumetric water content was monitored using a ThetaProbe ML3 sensor connected to an HH2 moisture meter (Delta-T Devices Ltd., Cambridge, UK). Irrigation events were triggered when soil water depletion reached approximately 40% of available water, corresponding to a volumetric water content of ~25%.

Fertilisation began 15 days after transplanting (DAT) with a foliar application of the fertiliser Rebrote (total N 10%, ammoniacal N 10%, P_2_O_5_ 50%, K_2_O 8%) at a concentration of 1.5 g L^−1^. Subsequent soil fertilisation was carried out using COMOS R^®^ as a source of NPK (14-8-19) and micronutrients (CaO 4.0%, MgO 2.0%, S 7%, B 0.08%, Cu 0.03%, Fe 0.17%, Mn 0.07%, Zn 0.16%), applied at 0.5 g L^−1^ by drench every five days.

Weed control and phytosanitary management were conducted following standard local agronomic recommendations and were kept consistent across treatments. These management practices ensured that observed differences among incident-PPFD regimes, genotypes, and locations primarily reflected the intended experimental factors rather than avoidable water or nutrient stress.

**Figure 9:**
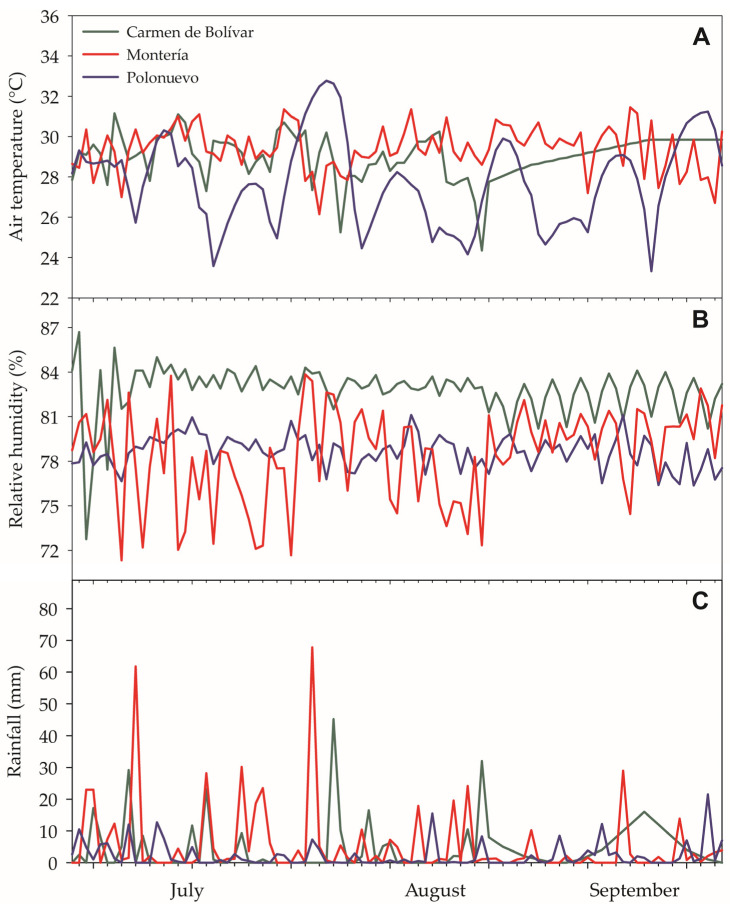
Daily meteorological conditions recorded during the experimental period (28 June–28 September 2024) at the three Caribbean locations: (**A**) air temperature (°C), (**B**) relative humidity (%), and (**C**) daily rainfall (mm). Meteorological data were collected using HOBO^®^ U23 Pro v2 sensors for temperature and relative humidity, and a HOBO^®^ RG3-M rain gauge for precipitation.

Air temperature and relative humidity were recorded daily under natural conditions using mini-weather stations equipped with outdoor sensors (HOBO^®^ U23 Pro v2), and precipitation was recorded using a calibrated HOBO^®^ RG3-M sensor with 0.2 mm rainfall-event resolution. During the experimental period, the mean temperature in Carmen de Bolívar was 29 °C, relative humidity was 76%, and accumulated precipitation was 419 mm. In Montería, the mean temperature was 29.3 °C, relative humidity was 78.6%, and accumulated precipitation was 508 mm. In Polonuevo, the mean temperature was 27.8 °C, with accumulated precipitation of 203 mm (Figure 9).

**Figure 10:**
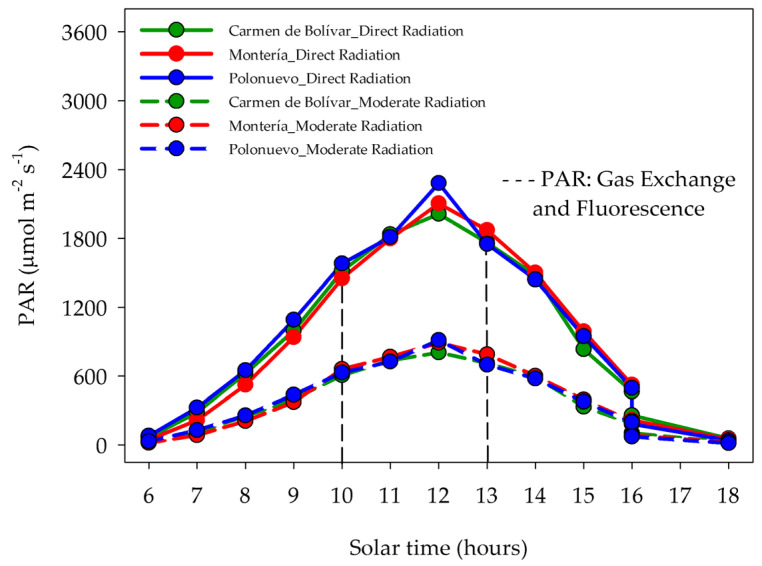
Diurnal course of incident photosynthetic photon flux density (PPFD; μmol photons m^−2^ s^−1^) measured under two incident-radiation environments: moderate radiation (60% transmittance shade net) and direct radiation (full exposure; 100% transmittance). PPFD was recorded hourly on a clear day from 06:00 to 18:00 h using a CI-340 quantum sensor (CID Inc., Washington, DC, USA). For treatment characterisation, representative PPFD levels were estimated as the mean between 10:00 and 13:00 h (moderate radiation: 600 μmol photons m^−2^ s^−1^; direct radiation: 1800 μmol photons m^−2^ s^−1^).

PPFD was quantified using a quantum sensor (CI-340 Photosynthesis Systems, CID Inc., Washington, DC, USA) under both radiation environments by collecting hourly records on a clear day from 06:00 to 18:00 h (Figure 10). For treatment characterisation, representative PPFD levels were estimated as the mean of measurements recorded between 10:00 and 13:00 h, under moderate radiation (600 μmol photons m^−2^ s^−1^) and direct radiation (1800 μmol photons m^−2^ s^−1^).

### 4.2. Gas-Exchange Measurements

At 85 days after transplanting (DAT), leaf gas exchange was measured on the third fully expanded, healthy, photosynthetically active leaf located in the upper canopy stratum of each *S. rebaudiana* genotype at each location, under each incident-radiation environment (600 and 1800 μmol photons m^−2^ s^−1^). Third, the study represents a single evaluation window 1800 μmol photons m^−2^ s^−1^), using a portable open-flow infrared gas analyser (LI-6800, LI-COR Biosciences, Lincoln, NE, USA), following Ariza-Gonzalez et al. [58] and Pompelli et al. [47]. Measurements were recorded between 10:00 and 13:00 h, under a fixed reference CO_2_ concentration of 400 μmol mol^−1^ (pure CO_2_ cartridge iSi, iSi inspiring solutions, Vienna, Austria), an air flow rate of 500 μmol s^−1^, and a leaf-chamber temperature of 28 °C. Net photosynthesis (*A_N_*, μmol CO_2_ m^−2^ s^−1^), stomatal conductance (*g_s_*, mmol H_2_O m^−2^ s^−1^), transpiration (*E*, mmol H_2_O m^−2^ s^−1^), and intercellular CO_2_ concentration (C_i_, μmol mol^−1^) were recorded. Water-use efficiency (WUE, μmol CO_2_ mmol^−1^ H_2_O) was calculated from *A_N_* and E (WUE = *A_N_* /E). This WUE therefore represents instantaneous (leaf-level) water-use efficiency derived from simultaneous gas-exchange measurements and does not represent whole-plant or seasonal water consumption. Because total irrigation volume per plant/plot was not recorded, whole-plant water consumption could not be quantified in this study.

### 4.3. Chlorophyll Fluorescence Measurements

Light-adapted chlorophyll fluorescence was assessed using the LI-6800 portable infrared gas analyser (LI-COR Biosciences, Lincoln, NE, USA) equipped with an integrated fluorescence chamber head. Measurements were performed at 85 DAT on the third fully expanded, healthy, photosynthetically active leaf located in the upper canopy stratum of each genotype, under each incident-radiation environment (600 and 1800 μmol photons m^−2^ s^−1^) and location, and within the same measurement window used for gas exchange (10:00–13:00 h). A saturating pulse of white light (6000 μmol photons m^−2^ s^−1^) was applied to determine maximum fluorescence in the light-adapted state (F_m′). Far-red illumination was then used to estimate minimal fluorescence in the light (F_o′), and steady-state fluorescence under actinic light (F_s) was recorded. From these fluorescence parameters, the effective quantum yield of PSII (ΦPSII), maximum efficiency of PSII in the light-adapted state (Fv′/Fm′ ratio), electron transport rate (ETR, μmol e^−^ m^−2^ s^−1^), and photochemical quenching coefficient (*qP*) were derived following Rodriguez-Paez et al. [16]. ETR was computed as ETR = ΦPSII × PPFD × 0.5 × 0.84, where PPFD is the incident photosynthetic photon flux density, 0.5 assumes equal excitation of photosystems I and II, and 0.84 is a constant leaf absorptance; therefore, ETR is interpreted as a comparative proxy across treatments rather than an absolute measure. Because leaf optical properties were not measured, potential variation in absorptance among genotypes or radiation regimes cannot be excluded.

### 4.4. Morphometric and Biomass Measurements

Biometric traits were quantified at harvest to characterise canopy development and biomass allocation. Plant height (PH, cm) was measured from the stem base to the apical meristem, and the number of leaves (NL) was counted per plant. Leaf dimensions (length, L, and width, W) were recorded for all leaves, and leaf area (LA, cm^2^) was estimated using the power equation LA = 0.2798 + (0.6341 × L × W), following Hernández-Fernández et al. [59]. The leaf area index (LAI; dimensionless) was then calculated from LA and ground area.

To quantify biomass components, plants were separated into roots, stems, and leaves, and root dry weight (RDW, g), stem dry weight (SDW, g), and leaf dry weight (LDW, g) were determined after oven drying. Plant fractions were dried in a forced-air oven (BD-240, Binder-Kasai, Tuttlingen, Germany) at 72 °C for 72 h until reaching a constant mass and weighed using an analytical balance. Total dry weight (TDW, g) was computed as the sum of RDW, SDW, and LDW.

### 4.5. Leaf Yield and Harvest Index Determination

Leaf yield and harvest index were quantified at 90 days after transplanting (DAT). Harvest was conducted simultaneously at the three locations using the central rows of each experimental unit (EU) to minimise border effects. Plants were cut and immediately defoliated, and fresh leaves were weighed to obtain leaf mass per EU. Leaf yield (Y, kg ha^−1^) was estimated by scaling leaf mass from the harvested EU area to a hectare, following Jarma et al. [14].

Harvest index (HI) was calculated to describe the efficiency of biomass allocation to the commercial organ. After oven drying, HI was computed as the ratio of leaf dry weight (commercial organ) to total plant dry weight, as described by Combatt-Caballero et al. [60]: HI = LDW/TDW; where LDW is leaf dry weight and TDW is total dry weight.

### 4.6. Experimental Design and Treatments

The study was conducted as a multi-environment field experiment across three Caribbean locations (El Carmen de Bolívar, Montería, and Polonuevo) under a split-plot randomised complete block design (RCBD) arranged as a 3 × 2 × 4 factorial (location × incident-radiation level × genotype). Location (three sites) was treated as a fixed factor. Within each location, incident radiation was assigned to the main plots (moderate radiation under shade net; 60% transmittance, and direct radiation under full exposure), operationally denoted as 600 and 1800 μmol photons m^−2^ s^−1^, respectively. Genotype was assigned to subplots within each main plot, comprising three experimental clones (L102, L020, and L082) and the commercial control (‘Morita II’). Treatment allocation followed standard split-plot randomisation: radiation environments were randomised within each block, and genotypes were randomised within each radiation main plot. Each experimental unit (subplot) consisted of 8 m^2^, and all treatments were replicated four times (four blocks per location). For statistical purposes, the subplot (genotype within radiation within block, at a given location) was considered the experimental unit for all response variables. Additional buffer (guard) plots were established around the analytical subplots to stabilise radiation environments and minimise edge effects; buffer plots were not included in the statistical analyses.

### 4.7. Statistical Analysis

All response variables were analysed under a split-plot randomised complete block design (RCBD) using a three-factor model including location (three levels: El Carmen de Bolívar, Montería and Polonuevo), incident radiation (two levels: moderate and direct), and genotype (four levels: L102, L020, L082 and ‘Morita II’), as well as all interactions. Within each location, incident radiation was assigned to main plots within blocks, and genotypes were randomised as subplots nested within each radiation main plot. Location, radiation and genotype were treated as fixed effects, and blocks were treated as random effects within each location.

For inference, the subplot (individual genotype subplot) was considered the experimental unit. Given the split-plot layout, ANOVA used the appropriate error strata, testing main-plot effects (radiation and location × radiation) against the main-plot error term (block × radiation within location), and testing subplot effects (genotype and interactions involving genotype) against the residual (subplot) error. When significant effects were detected, mean separation was performed using Tukey’s HSD at *p* < 0.05. Analyses were conducted in SAS v.9.4 (SAS Institute Inc., Cary, NC, USA).

Graphical outputs were produced using SigmaPlot v.14.0 (Systat Software, Inc., San José, CA, USA). Pearson correlation analysis and principal component analysis (PCA) were performed in R v.4.5.1 (R Foundation for Statistical Computing, Vienna, Austria), using the packages psych, Hmisc, FactoMineR, factoextra and circlize. The complete dataset used for these analyses is provided in Table S4.

## 5. Conclusions

Across three Caribbean field environments, the effects of incident radiation on *Stevia rebaudiana* were strongly contingent on site conditions and genotype, revealing clear genotype × environment structure in both physiology and yield. Direct radiation enhanced *A_N_* and related gas-exchange traits at Carmen de Bolívar and Polonuevo but reduced *A_N_* at Montería, while fluorescence responses showed site-specific shifts in PSII functioning (ΦPSII, ETR, Fv′/Fm′ and *qP*). Agronomic performance likewise depended on the interaction of site, radiation and genotype: mean leaf yield was highest at Carmen de Bolívar, intermediate at Montería and lowest at Polonuevo, with L102 (and frequently L020 and ‘Morita II’) outperforming L082 across environments. Complementary correlation and PCA analyses supported coordinated associations between gas exchange, photochemical traits, and productive performance across radiation and location contexts. Collectively, these results support environment-specific light management combined with targeted genotype deployment as a practical strategy to optimise *S. rebaudiana* leaf yield under tropical field conditions while highlighting L082 as consistently underperforming in the tested environments.

## Figures and Tables

**Table 1:** Plant height and leaf area under contrasting incident radiation environments and across genotypes of *Stevia rebaudiana* in three Caribbean locations (Colombia). Different uppercase letters indicate significant differences between radiation levels (upper section) and among genotypes (lower section) within the same location; different lowercase letters indicate significant differences among locations within the same radiation level (upper section) and within the same genotype (lower section). Values are means (±SE). *n* = 16.

Radiation	Plant Height (cm)			Leaf Area (cm^2^)		
	Carmen de Bolívar	Montería	Polonuevo	Carmen de Bolívar	Montería	Polonuevo
600 μmol photons m^−2^ s^−1^	47.93 ± 2.85 Ac	65.37 ± 4.70 Aa	52.74 ± 3.77 Ab	457.70 ± 52.27 Ac	1488.46 ± 209.88 Aa	1227.77 ± 150.14 Ab
1800 μmol photons m^−2^ s^−1^	49.57 ± 2.91 Ab	56.11 ± 4.09 Ba	41.67 ± 2.91 Bc	577.33 ± 78.04 Ac	1441.99 ± 190.67 Aa	807.05 ± 86.78 Bb
**Genotype**	**Plant Height (cm)**			**Leaf Area (cm^2^)**		
	**Carmen de Bolívar**	**Montería**	**Polonuevo**	**Carmen de Bolívar**	**Montería**	**Polonuevo**
L020	54.21 ± 1.13 Ab	68.65 ± 2.08 Aa	57.05 ± 2.03 Ab	654.59 ± 39.16 Ac	2043.60 ± 86.60 Aa	1204.82 ± 113.29 Ab
L082	30.35 ± 0.38 Ba	32.06 ± 0.93 Ba	26.20 ± 0.83 Cb	119.25 ± 2.96 Cb	293.44 ± 23.06 Ca	332.21 ± 19.38 Ca
L102	56.03 ± 0.71 Ab	71.13 ± 1.70 Aa	52.66 ± 1.85 Bb	590.14 ± 23.43 Bc	1812.09 ± 104.52 Ba	1334.19 ± 95.92 Ab
Morita II	54.40 ± 1.18 Ab	71.12 ± 1.63 Aa	52.90 ± 2.48 Bb	706.07 ± 48.12 Ac	1711.77 ± 136.35 Ba	1198.41 ± 95.49 Ab

**Table 2:** Leaf area index, number of leaves, root dry weight, stem dry weight, leaf dry weight and total dry weight of four *Stevia rebaudiana* genotypes under contrasting radiation environments across three Caribbean locations (Colombia). Different uppercase letters indicate significant differences between radiation levels within the same location and genotype; different lowercase letters indicate significant differences among genotypes within the same radiation level and location; asterisks (*) indicate significant differences among locations within the same radiation level and genotype. Values are means (±SE). *n* = 4.

Genotype	Leaf Area Index					
	Carmen de Bolívar		Montería		Polonuevo	
	600 μmol Photons m^−2^ s^−1^	1800 μmol Photons m^−2^ s^−1^	600 μmol Photons m^−2^ s^−1^	1800 μmol Photons m^−2^ s^−1^	600 μmol Photons m^−2^ s^−1^	1800 μmol Photons m^−2^ s^−1^
L020	1.29 ± 0.16 Aa	1.26 ± 0.04 Aa	3.86 ± 0.11 Ab *	4.32 ± 0.72 Ba *	0.94 ± 0.15 Aa	0.57 ± 0.05 Ab
L082	0.17 ± 0.01 Ba	0.20 ± 0.04 Ba	0.77 ± 0.05 Ca *	0.72 ± 0.01 Ca *	0.21 ± 0.03 Ba	0.20 ± 0.02 Ba
L102	1.08 ± 0.19 Aa	1.19 ± 0.03 Aa	3.31 ± 0.24 Bb *	4.07 ± 0.22 Ba *	0.98 ± 0.09 Aa	0.68 ± 0.11 Ab
Morita II	1.19 ± 0.13 Aa	0.95 ± 0.20 Aa	3.50 ± 0.30 Ab *	5.32 ± 0.45 Aa *	0.94 ± 0.49 Aa	0.56 ± 0.03 Ab
**Genotype**	**Number of Leaves**					
	**Carmen de Bolívar**		**Montería**		**Polonuevo**	
	**600 μmol photons m^−2^ s^−1^**	**1800 μmol photons m^−2^ s^−1^**	**600 μmol photons m^−2^ s^−1^**	**1800 μmol photons m^−2^ s^−1^**	**600 μmol photons m^−2^ s^−1^**	**1800 μmol photons m^−2^ s^−1^**
L020	288 ± 1.23 Aa	258 ± 6.49 Bb	942 ± 22.19 Ab *	1339 ± 208.73 Aa *	240 ± 26.89 Aa	239 ± 26.51 Aa
L082	70 ± 1.18 Ba	64 ± 1.80 Ca	281 ± 10.38 Ca *	250 ± 6.87 Cb *	108 ± 10.47 Ca	118 ± 5.52 Ca
L102	290 ± 9.04 Aa	262 ± 4.57 Bb	934 ± 42.95 Ab *	1241 ± 63.59 Ba *	262 ± 10.49 Aa	251 ± 38.68 Aa
Morita II	284 ± 5.45 Aa	297 ± 3.45 Aa	809 ± 42.83 Bb *	1212 ± 101.64 Ba *	177 ± 17.58 Ba	149 ± 7.96 Bb
**Genotype**	**Root Dry Weight (g)**					
	**Carmen de Bolívar**		**Montería**		**Polonuevo**	
	**600 μmol photons m^−2^ s^−1^**	**1800 μmol photons m^−2^ s^−1^**	**600 μmol photons m^−2^ s^−1^**	**1800 μmol photons m^−2^ s^−1^**	**600 μmol photons m^−2^ s^−1^**	**1800 μmol photons m^−2^ s^−1^**
L020	9.83 ± 1.31 Bb *	14.11 ± 2.56 Aa	5.25 ± 0.51 Ab	16.66 ± 3.02 Aa *	6.40 ± 0.88 Ab	8.58 ± 1.85 Aa
L082	11.25 ± 2.99 Aa *	7.63 ± 1.74 Cb	3.72 ± 0.74 Bb	10.26 ± 2.37 Ca *	6.38 ± 0.63 Aa	3.55 ± 0.66 Db
L102	14.60 ± 2.44 Aa *	9.71 ± 2.27 Bb	5.95 ± 0.84 Aa	14.74 ± 5.36 Ba *	5.63 ± 0.93 Bb	7.23 ± 1.52 Ba
Morita II	8.49 ± 1.76 Bb *	15.83 ± 1.16 Aa *	3.78 ± 0.51 Bb	14.46 ± 2.54 Ba	5.40 ± 0.78 Ba	5.28 ± 1.67 Ca
**Genotype**	**Stem Dry Weight (g)**					
	**Carmen de Bolívar**		**Montería**		**Polonuevo**	
	**600 μmol photons m^−2^ s^−1^**	**1800 μmol photons m^−2^ s^−1^**	**600 μmol photons m^−2^ s^−1^**	**1800 μmol photons m^−2^ s^−1^**	**600 μmol photons m^−2^ s^−1^**	**1800 μmol photons m^−2^ s^−1^**
L020	46.17 ± 3.61 Bb *	61.51 ± 8.98 Ba *	27.26 ± 3.03 Bb	50.36 ± 2.23 Ca	18.48 ± 2.64 Aa	14.80 ± 1.12 Bb
L082	21.44 ± 2.61 Da *	20.00 ± 1.47 Da *	9.77 ± 1.92 Cb	15.02 ± 1.26 Da	5.85 ± 1.07 Db	7.35 ± 1.46 Ca
L102	51.44 ± 4.33 Ab *	71.52 ± 5.88 Aa *	39.26 ± 4.07 Aa	58.76 ± 9.07 Ba	15.60 ± 3.23 Bb	20.00 ± 2.85 Aa
Morita II	40.96 ± 3.91 Ca *	46.83 ± 3.54 Ca *	26.20 ± 3.73 Bb	68.74 ± 3.49 Aa	12.33 ± 0.86 Cb	14.70 ± 2.93 Ba
**Genotype**	**Leaf Dry Weight (g)**					
	**Carmen de Bolívar**		**Montería**		**Polonuevo**	
	**600 μmol photons m^−2^ s^−1^**	**1800 μmol photons m^−2^ s^−1^**	**600 μmol photons m^−2^ s^−1^**	**1800 μmol photons m^−2^ s^−1^**	**600 μmol photons m^−2^ s^−1^**	**1800 μmol photons m^−2^ s^−1^**
L020	42.12 ± 3.86 Ab *	101.16 ± 18.90 Aa *	24.75 ± 2.71 Bb	64.14 ± 2.88 Ba	15.43 ± 1.99 Ab	18.20 ± 1.10 Aa
L082	19.28 ± 1.97 Ba *	18.64 ± 0.61 Ca *	8.10 ± 1.16 Cb	11.78 ± 0.94 Ca	4.38 ± 0.63 Cb	7.18 ± 3.02 Da
L102	44.42 ± 4.64 Ab *	103.48 ± 15.64 Aa *	34.77 ± 5.39 Ab	70.33 ± 11.81 Ba	9.38 ± 1.99 Bb	16.68 ± 1.37 Ba
Morita II	40.07 ± 3.91 Ab *	83.68 ± 13.73 Ba *	25.99 ± 5.55 Bb	87.76 ± 7.30 Aa	12.45 ± 1.43 Ab	15.00 ± 3.70 Ca
**Genotype**	**Total Dry Weight (g)**					
	**Carmen de Bolívar**		**Montería**		**Polonuevo**	
	**600 μmol photons m^−2^ s^−1^**	**1800 μmol photons m^−2^ s^−1^**	**600 μmol photons m^−2^ s^−1^**	**1800 μmol photons m^−2^ s^−1^**	**600 μmol photons m^−2^ s^−1^**	**1800 μmol photons m^−2^ s^−1^**
L020	98.12 ± 6.64 Bb *	176.77 ± 24.12 Aa *	57.26 ± 5.48 Bb	131.16 ± 5.40 Ba	40.30 ± 5.44 Aa	41.58 ± 3.35 Aa
L082	51.97 ± 6.71 Ca *	46.27 ± 3.35 Ca *	21.59 ± 3.57 Cb	37.05 ± 4.43 Ca	16.60 ± 2.28 Ca	18.08 ± 4.04 Ca
L102	110.46 ± 10.52 Ab *	184.71 ± 17.36 Aa *	79.98 ± 9.93 Ab	143.82 ± 24.95 Ba	30.60 ± 6.12 Bb	43.90 ± 4.70 Aa
Morita II	89.52 ± 8.60 Bb *	146.34 ± 18.09 Ba *	55.98 ± 9.63 Bb	170.96 ± 12.55 Aa	30.18 ± 2.85 Ba	34.98 ± 7.66 Ba

**Table 3:** Substrate chemical characteristics.

Location	pH	O.C	S	P	Ca	Mg	K	Na	Cu	Fe	Mn	B
	1:1	%	mg kg^−1^		cmol (+) kg^−1^				mg kg^−1^			
Carmen de Bolívar	6.98	2.28	10.2	35.63	28.1	5.35	1.14	0.26	3.59	18.02	1.26	0.75
Montería	6.34	0.75	16.4	36.0	2.91	1.36	0.46	0.20	0.69	12.9	14.8	0.32
Polonuevo	6.97	0.63	17.0	14.0	3.64	0.73	0.079	0.23	0.30	43.0	72.0	0.04

O.C = Organic Carbon; S = Sulfur; P = Phosphorus; Ca = Calcium; Mg = Magnesium; K = Potassium; Na = Sodium; Cu = Copper; Fe = Iron; Mn = Manganese; B = Boron.

## Data Availability

The original data supporting the findings of this study are available in the Supplementary Files accompanying this article, including Table S1 (ANOVA mean squares), Table S2 (Pearson correlation matrix), Table S3 (PCA loadings, contributions, and explained variance), and Table S4 (complete dataset). Further inquiries can be directed to the corresponding author.

## Supplementary Materials

The following supporting information can be downloaded at: https://www.mdpi.com/article/10.3390/plants15060896/s1, Table S1: three-way ANOVA mean squares (site, radiation, genotype and interactions); Table S2: Pearson correlation matrix among all measured variables; Table S3: PCA loadings, contributions and explained variance; Table S4: Data Stevia.

## Abbreviations

The following abbreviations are used in this manuscript:

AMMI	Additive main effects and multiplicative interaction
*A_N_*	Net photosynthetic rate (net CO_2_ assimilation rate)
ANOVA	Analysis of variance
C_i_	Intercellular CO_2_ concentration
DAT	Days after transplanting
*E*	Transpiration
ETR	Electron transport rate
EU	Experimental unit
Fv′/Fm′	Maximum efficiency of PSII in the light-adapted state
GEI	Genotype × environment interaction
*g**_s_*	Stomatal conductance
GGE	Genotype plus genotype-by-environment interaction
HI	Harvest index
HPLC	High-performance liquid chromatography
HSD	Honestly significant difference
LA	Leaf area
LAI	Leaf area index
LDW	Leaf dry weight
L_s_	Stomatal limitation
NL	Number of leaves
NPQ	Non-photochemical quenching
PAR	Photosynthetically active radiation
PCA	Principal component analysis
PH	Plant height
PPFD	Photosynthetic photon flux density
PSII	Photosystem II
*qP*	Photochemical quenching coefficient
RCBD	Randomised complete block design
RDW	Root dry weight
SDW	Stem dry weight
SE	Standard error
TDW	Total dry weight
VPD	Vapour pressure deficit
WUE	Water-use efficiency
Y	Leaf yield
ΦPSII	Effective quantum yield of PSII
